# Healthcare resource utilization and costs in immunodeficient patients receiving subcutaneous Ig: Real-world evidence from France

**DOI:** 10.1371/journal.pone.0313694

**Published:** 2025-01-24

**Authors:** Guillaume Lefèvre, Isabelle Borget, Cinira Lefèvre, Chahrazed Maherzi, Arnaud Nucit, Mouna Hennaoui, Aurélie Schmidt, Hannah Lennon, Benjamin Grenier, Florent Daydé, Nizar Mahlaoui

**Affiliations:** 1 Institute of Immunology, Institute for Translational Research in Inflammation (Infinite ‐ U1286), University of Lille, CHU Lille, Inserm, Lille, France; 2 Department of Biostatistics and Epidemiology, Gustave Roussy, Paris-Saclay University, Gif-sur-Yvette, Villejuif, France; 3 Oncostat ‐ U1018, Inserm, Paris-Saclay University, “Ligue Contre le Cancer” Labeled Team, Gif-sur-Yvette, Villejuif, France; 4 GRADES, Paris-Saclay University, Gif-sur-Yvette, Châtenay-Malabry, France; 5 Takeda France, Paris, France; 6 HEVA, Lyon, France; 7 French National Reference Center for Primary Immunodeficiencies (CEREDIH) and Pediatric Immunology, Hematology and Rheumatology Unit, Necker Enfants Malades University Hospital, Assistance Publique ‐ Hôpitaux de Paris (APHP), Paris, France; The University of Hong Kong, HONG KONG

## Abstract

**Background:**

Subcutaneous immunoglobulin (SCIg) replacement therapy is indicated for patients with hypogammaglobulinemia caused by primary (PID) and secondary immunodeficiencies (SID).

**Objective:**

To compare healthcare resource utilization (HCRU) and related direct medical costs of patients in France treated with weekly conventional SCIg (cSCIg) vs monthly hyaluronidase-facilitated SCIg (fSCIg).

**Methods:**

This retrospective study of Ig-naïve patients with PID or SID newly receiving a SCIg between 2016 and 2018, extracted from the French National Healthcare reimbursement database (SNDS), analyzed the SCIg-related HCRU and reimbursed costs generated from in-hospital (hospitalizations and SCIg doses) or at-home (nurse visits [NV] and pump provider visits [PPV], drug doses) SCIg administration.

**Results:**

Overall, 2,012 patients (PID:534; SID:1,478) were analyzed. The follow-up duration varied between 7.5 and 8.7 months according to sub-groups. Compared with fSCIg-treated patients, monthly mean rates of NV and PPV were respectively 2.5 and 3.1 times higher in PID, and 1.6 and 3.1 times higher in SID cSCIg-treated patients. Monthly mean rates for SCIg administration-related hospitalizations were lower overall, while their costs were 1.6 and 1.8 times higher for cSCIg than fSCIg subgroups, in PIDs and SIDs respectively; these results are due to more frequent hospitalizations with fSCIg being mainly shorter, without stayover. Total HCRU costs from the French NHI’s perspective were estimated to be lower with fSCIg vs cSCIg, in PIDs and SIDs.

**Conclusion:**

This study provides real-world evidence of SCIg administration in a large French population. Patients with PID or SID treated with fSCIg had fewer at-home HCRU and lower overall costs for in-hospital or at-home SCIg administration compared with cSCIg-treated patients.

## Introduction

Immunodeficiencies, a group of pathologies related to insufficiencies of one or more immunological functions, are primarily classified into primary immunodeficiencies (PIDs) if they are of genetic origin or without identified underlying disease, or secondary immunodeficiencies (SIDs) if they are related to, but not exclusively, hematological malignancies or drug-induced [1,2]. PIDs are a group of more than 485 inherited conditions [3] affecting the immune system, leading to increased susceptibility to infections and non-infectious complications [4]. The French national Reference Center for PID (CEREDIH) Registry currently is the largest national registry with more than 8,700 patients with PID and [5] has estimated the overall prevalence of PID to be more than 11 per 100,000 inhabitants [5,6]. However, underdiagnosis of PIDs has led to an underestimated prevalence reported in national and regional registries [6]. SIDs caused by a medical condition (for example in chronic lymphocytic leukemia [CLL], multiple myeloma [MM], or pre- and post-allogeneic hematopoietic stem-cell transplantation [HSCT]) or certain medications are more common than PIDs and result in recurring infections, potentially leading to end-organ damage and mortality [7,8].

PID and SID patients with hypogammaglobulinemia receive long-term immunoglobulin (Ig) replacement therapy using either intravenous Ig (IVIg) or subcutaneous Ig (SCIg) administration [9]. In patients with hypogammaglobulinemia [10], rigorous Ig-replacement therapy (IgRT) is recommended [11,12]. Conventional SCIgs (cSCIgs) indicated for the treatment of PID or SID (in CLL, MM and pre/post-HSCT) can be self-administered at home once weekly [13–15]. Facilitated SCIg (fSCIg) is a dual vial unit of 10% IgG and recombinant human hyaluronidase. The hyaluronidase component temporarily depolymerizes hyaluronan to allow large volumes of Ig to be administered subcutaneously once every 3-4 weeks at a single infusion site [16]. fSCIg has been approved in Europe for the treatment of PID and SID since May 2013 [17]. fSCIg was launched in France in these indications in December 2016 and is reimbursed in extended indication of SID situations in France since May 2021 [18,19].

cSCIg and fSCIg replacement therapies are effective and well tolerated in patients with PID and SID [13,20–26]. Few burden of illness studies suggested that home-based SCIg administration is less expensive and convenient compared with IVIg administration in PID treatment [27,28]. Healthcare resource utilization (HCRU) for at-home SCIg administration was 25%-75% less costly than that for in-hospital IVIg administration in Europe [29]. In France, estimated treatment costs, based on a questionnaire answered by a cohort of patients with congenital agammaglobulinemia or hyper-IgM syndrome, were higher for IVIg vs SCIg [30]. Along with the site of care, the overall cost of mean Ig doses per patient was higher for IVIg vs SCIg [30,31].

As SCIg’s in hospital or at home can be administered at different frequencies, it is relevant to investigate if such differences have an impact on HCRU and associated costs. Moreover, home-based SCIg infusions can be either administered by a nurse or self-administered, and both require service provider visits for infusion pump installation/monitoring and ancillaries. Therefore, the present study compared the HCRU and related costs of in-hospital and at-home administration of monthly fSCIg vs those of weekly cSCIgs in patients with PID and SID, using exhaustive data from the French National healthcare reimbursement database, *système national des données de santé* (SNDS) [32].

## Materials and methods

### Study design

**Fig 1:**
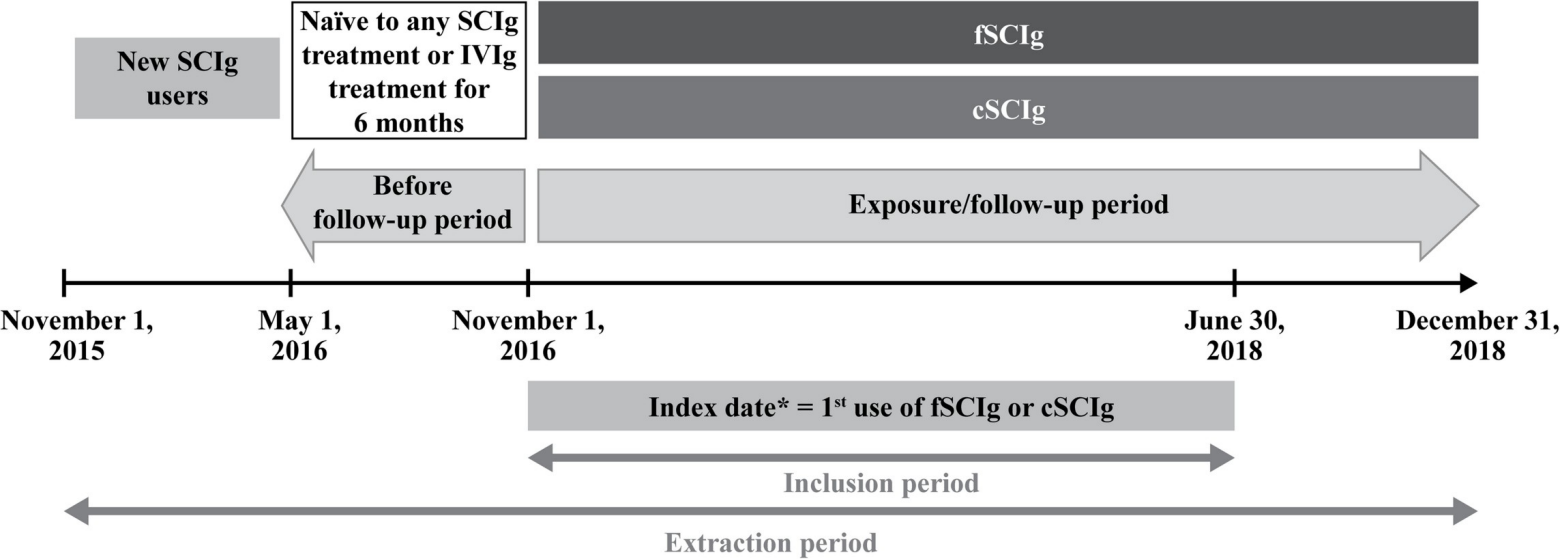
Study design. The study consisted of three key periods: inclusion period (November 1, 2016, to June 30, 2018), follow-up period (index date to December 31, 2018), and extraction period (November 1, 2015, to December 31, 2018). *The index date was defined as the first use of any SCIg within the inclusion period. cSCIg-1: Hizentra^®^; cSCIg-2: Gammanorm^®^; fSCIg: Hyqvia^®^ cSCIg, conventional SCIg; fSCIg, facilitated SCIg; IVIg, intravenous immunoglobulin; SCIg, subcutaneous immunoglobulin.

This was a retrospective observational cohort study conducted in French patients newly treated (no Ig-administration during the previous 6 months) with fSCIg or cSCIg for the treatment of PID or SID. The entire study period or extraction period extended from November 1, 2015, to December 31, 2018. The inclusion period extended from November 1, 2016, to June 30, 2018, to include patients who did not receive any Ig treatment in the past 6 months. The index date was defined as the date of the first dispensation, during the inclusion period, of any SCIg marketed in France over the study period (HyQvia^®^ [Baxalta Innovations GmbH], Hizentra^®^ [CSL Behring AG, Bern, Switzerland], or Gammanorm^®^ [Octapharma AB, Stockholm, Sweden]). Patients were followed up from the index date until the end of the study period (December 31, 2018), death, treatment switch, treatment discontinuation (without evidence of switch), or loss to follow-up, whichever occurred first (Fig 1).

Patient consent was not necessary since secondary data have been collected. The study protocol was approved by the Ethical and Scientific Committee for Research, Studies and Evaluations in the field of Health (*Comité éthique et scientifique pour les recherches*, *les études et les évaluations dans le domaine de la santé* [CESREES]; TPS file 1204887, authorization granted on January 17, 2020), and authorization to use these data was granted by the French Data Protection Authority (*Commission nationale de l’informatique et des libertés* [CNIL]; decision DR-2020-043, authorization number 919332v1 granted on January 31, 2020).

### Data sources

This study used data from SNDS French public bodies, including *Système national d’information interrégimes de l’Assurance Maladie* (SNIIRAM) [33], a database that contains individual-level data for healthcare resource utilization and reimbursed costs of patients care. The database uses social security numbers of patients as unique, anonymous identifiers, allowing for the data capture of individual-level healthcare claims of over 99% of the French population [34].

In accordance with French regulations, patient consent was not necessary because this study was based on secondary data with the public health interest in assessing the clinical management and related costs within PID and SID treated patients in France, while ensuring protection of patients’ rights and freedom.

The authorization to use the data was granted by the French data protection authority (Commission Nationale de l’Informatique et des Libertés, CNIL) (Decision DR-2019-277, and authorization No. 919332) and the study protocol obtained approval from the committee for research, studies, and evaluations in the field of health (Comité d’expertise pour les recherches, les études et les évaluations dans le domaine de la santé, CEREES) (Decision TPS 712802). Data was first accessed on January 31, 2020, following the authorizations granted by CESREES and CNIL.

### Study population

The patient cohort consisted of SCIg users (adult and pediatric) not treated with any SCIg or IVIg within 6 months before their inclusion in the study. The PID cohort included patients with a long-term disease status (Affections de longue durée [ALD]) [35] and/or hospital stays with a PID diagnosis, and the SID cohort included patients who did not have any PID-related code. Patients with nonunique identifiers in the SNDS database (including twins, multiple births, and fictive identifiers) and patients with data inconsistencies, patients who had different SCIgs delivered at the index date, and patients with chronic inflammatory demyelinating polyneuropathy (International Classification of Diseases, Tenth Revision [ICD-10] code G618) or an immunomodulatory indication at any time during the follow-up period were excluded.

### Outcomes

Monthly HCRU was restricted to the resources related to SCIg-administration both in hospital and at home including pump provider visits (PPV) and private nurse visits (NV) and delivered SCIg doses. Mean related cost per patient for each HCRU was also evaluated.

### Assessments

Hospitalization-related HCRU for SCIg administration was assessed based on all hospitalizations for which SCIg delivery was identified through the Expensive Drugs List (*Liste en sus*) [36]. Home-related HCRU for SCIg administration included: (a) all services provided through service providers for pump installation and regular use of home perfusion (PERFADOM), which consisted of three packages (installation [PERFADOM 1], follow-up [PERFADOM 7], and consumables and ancillary packages [PERFADOM 10 and 25]), and (b) private nurses provided through private nursing procedures, reimbursable by health insurance. SCIg doses (g/month) delivered in-hospital or at home were calculated by the number of SCIg packs delivered and their dosages. HCRU related to the primary disease leading to Ig use was not part of the study scope.

All costs related to HCRU were analyzed from the perspective of health insurance and discounted to €2019 prices. Cost for in-hospital SCIg administration were based on the sum of all hospitalization costs, including the cost of hospital-administered expensive drugs and medical devices. For at-home SCIg administration, costs for PPV and NV were based on the costs identified in the databases. Cost for SCIg doses delivered after exclusion of those <8 g/month (non-clinically plausible doses) in hospital or at home were based on recalculated costs of Ig packs delivered (identified number of units of SCIg treatment multiplied by the unit cost). Pricing was implemented by the decree of April 12, 2016, or March 16, 2018, based on the study period.

Other variables measured included sex; age; affiliation to the complementary universal health coverage scheme for low-income people (*Couverture Maladie Universelle Complémentaire* [CMU-C]) [37]; comorbidities (cancer, cardiovascular diseases, diabetes mellitus, hypertension, and respiratory disorders); delivery of comedications classified by the Anatomical Therapeutic Chemical (ATC) class [38]; ALD status [35]; treatment switch, defined as having two consecutive dispensations of another type of Ig; and treatment discontinuation, defined as ≥2 months (PID cohort) or ≥6 months (SID cohort) without SCIg therapy (see S1 Fig in the Online Repository at www.jacionline.org).

### Statistical analyses

Descriptive statistics were used to display the frequency and mean monthly rate of each HCRU according to the type of indication. Differences in the mean monthly number of hospitalizations for SCIg administration with or without stayover during the entire study period, and within the first month and during the follow-up period were calculated. The mean number of each HCRU outcome during the follow-up period was compared between cSCIg- and fSCIg-treated patients using a Poisson regression model [39], which modeled counting data for quantifying the relative risk (RR; interpreted as the ratio of the mean number of each HCRU outcome in the cSCIg group to that in the fSCIg group) and its corresponding 95% confidence interval (CI). Mean SCIg dose was compared between cSCIg- and fSCIg-treated patients using linear regression models [40], which quantified the average difference between the mean doses of cSCIg and fSCIg. Mean monthly cost per patient for SCIg administration was analyzed using a gamma regression model (expressed as RR), which quantified continuous-valued and right-skewed data [41]. The covariates selected to be included in the initial model (sex, age, CMU-C, ALD status, co-treatments and comorbidities) were identified based on clinical input, and those with *P*<0.05 were retained in the final model. All analyses were conducted using SAS^®^ version 9.4.

## Results

### Patient disposition and baseline characteristics

**Fig 2:**
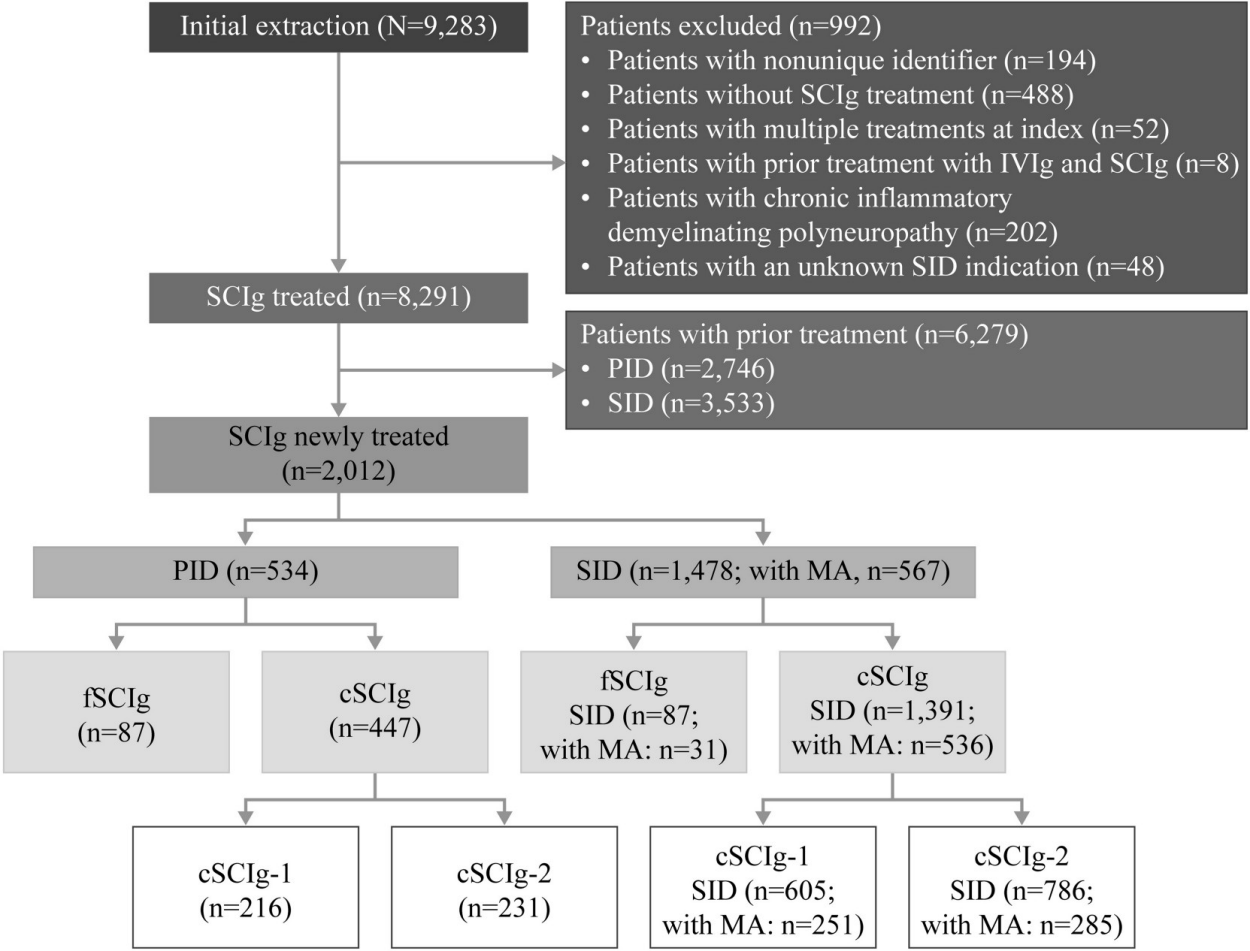
Patient disposition. cSCIg-1: Gammanorm^®^; cSCIg-2: Hizentra^®^; fSCIg: HyQvia^®^ cSCIg, conventional SCIg; fSCIg, facilitated SCIg; IVIg, intravenous immunoglobulin; MA, marketing authorization; PID, primary immunodeficiency; SCIg, subcutaneous immunoglobulin; SID, secondary immunodeficiency.

Of 9,283 patients extracted, 8,291 were identified as treated with SCIg. After excluding patients who had received prior treatment with IVIg or SCIg, 2,012 patients (PID: 534; SID: 1,478) newly treated (no Ig-administration during the previous 6 months) with fSCIg or cSCIg were included (Fig 2).

#### PID population

Mean age of fSCIg- and cSCIg-treated patients was 53.7 vs 53.6 years, respectively, and 55.2% vs 58.2% of patients were female (Table 1). The most frequent comedications were those treating digestive and metabolic disorders (35.6% vs 49.9%) and systemic antibacterials (40.2% vs 42.3%), and the most common comorbidities were respiratory disorders (36.8% vs 48.3%), cancer (31.0% vs 40.7%), and hypertension (15.0% vs 23.5%) in fSCIg- vs cSCIg-treated patients, respectively. Mean±SD follow-up duration was slightly lower in fSCIg-treated patients (7.7±5.3 months) than in cSCIg-treated patients (8.7±7.0 months). The main reason for follow-up censoring was treatment discontinuation in 51.7% vs 55.9% of fSCIg- vs cSCIg-treated patients, respectively.

**Table 1:** Demographics and baseline patient characteristics.

	PID				SID			
	fSCIg	cSCIg-1	cSCIg-2	cSCIg (all)	fSCIg	cSCIg-1	cSCIg-2	cSCIg (all)
	n = 87	n = 216	n = 231	n = 447	n = 87	n = 605	n = 786	n = 1,391
Age (years) at index date, mean±SD	54 ±16.88	55 ±21.99	52 ±23.09	54 ±22.59	68 ±12.76	65 ±15.80	65 ±16.59	65 ±16.25
Sex (female), n (%)	48 (55.17)	124 (57.41)	136 (58.87)	260 (58.17)	40 (45.98)	289 (47.77)	388 (49.36)	677 (48.67)
Affiliation to the CMU-C at index date, n (%)	3 (3.45)	7 (3.24)	16 (6.93)	23 (5.15)	1 (1.15)	28 (4.63)	21 (2.67)	49 (3.52)
Comedications (ATC classification) with ≥1 delivery for treatment, n (%)								
Alimentary tract and metabolism (A)	31 (35.63)	101 (46.76)	122 (52.81)	223 (49.89)	51 (58.62)	360 (59.50)	428 (54.45)	788 (56.65)
Antibacterial for systemic use (J101)	35 (40.23)	104 (48.15)	85 (36.80)	189 (42.28)	41 (47.13)	320 (52.89)	341 (43.38)	661 (47.52)
Drugs for obstructive airway diseases (R03)	16 (18.39)	56 (25.93)	55 (23.81)	111 (24.83)	18 (20.69)	96 (15.87)	146 (18.58)	242 (17.40)
Corticosteroids for systemic use (H02)	12 (13.79)	41 (18.98)	50 (21.65)	91 (20.36)	14 (16.09)	160 (26.45)	184 (23.41)	344 (24.73)
Musculoskeletal system (M)	7 (8.05)	31 (14.35)	36 (15.58)	67 (14.99)	15 (17.24)	98 (16.20)	91 (11.58)	189 (13.59)
Immunosuppressants (L04)	3 (3.45)	16 (7.41)	21 (9.09)	37 (8.28)	3 (3.45)	74 (12.23)	80 (10.18)	154 (11/07)
Antineoplastic agents (L01)	7 (8.05)	19 (8.80)	12 (5.19)	31 (6.94)	20 (22.99)	98 (16.20)	129 (16.41)	227 (16.32)
Antimycotics for systemic use (J02A)	2 (2.30)	7 (3.24)	10 (4.33)	17 (3.80)	2 (2.30)	30 (4.96)	32 (4.07)	62 (4.46)
Influenza vaccines (J07BB)	2 (2.30)	7 (3.24)	10 (4.33)	17 (3.80)	4 (4.60)	39 (6.45)	61 (7.76)	100 (7.19)
Antiprotozoals (P01)	1 (1.15)	3 (1.39)	7 (3.03)	10 (2.24)	4 (4.60)	32 (5.29)	43 (5.47)	75 (5.39)
Immunostimulants (L03)	2 (2.30)	3 (1.39)	6 (2.60)	9 (2.01)	6 (6.90)	25 (4.13)	31 (3.94)	56 (4.03)
Inclusion in a university hospital, n (%)	48 (55.17)	106 (49.07)	102 (44.16)	208 (46.53)	33 (37.93)	288 (47.60)	315 (40.08)	603 (43.35)
Presence of a long-term disease,* n (%)	40 (45.98)	67 (31.02)	80 (34.63)	147 (32.89)	73 (83.91)	542 (89.59)	725 (92.24)	1,267 (91.09)
Comorbidities, n (%)								
Cancer	27 (31.03)	93 (43.06)	89 (38.53)	182 (40.72)	73 (83.91)	456 (75.37)	640 (81.42)	1,096 (78.79)
Cardiovascular diseases	10 (11.49)	39 (18.06)	39 (16.88)	78 (17.45)	18 (20.69)	161 (26.61)	213 (27.10)	374 (26.89)
Diabetes mellitus	5 (5.75)	20 (9.26)	24 (10.39)	44 (9.84)	12 (13.79)	83 (13.72)	125 (15.90)	208 (14.95)
Hypertension	13 (14.94)	57 (26.39)	48 (20.78)	105 (23.49)	20 (22.99)	186 (30.74)	247 (31.42)	433 (31.13)
Respiratory disorders	32 (36.78)	103 (47.69)	113 (48.92)	216 (48.32)	38 (43.68)	201 (33.22)	269 (34.22)	470 (33.79)
Length of follow-up (months), mean±SD	7.72±5.30	8.24±6.80	9.03±7.22	8.65±7.03	7.53±4.58	8.79±6.73	8.08±6.63	8.39±6.68
Reasons for censorship, n (%)								
Discontinuation of treatment	45 (51.72)	121 (56.02)	129 (55.84)	250 (55.93)	25 (28.74)	281 (46.45)	431 (54.83)	712 (51.19)
Switch of treatment	5 (5.75)	12 (5.56)	20 (8.66)	32 (7.16)	7 (8.05)	27 (4.46)	54 (6.87)	81 (5.82)
Death	0 (0.00)	6 (2.78)	6 (2.60)	12 (2.68)	2 (2.30)	45 (7.44)	73 (9.29)	118 (8.48)
End of follow-up period	37 (42.53)	77 (35.65)	76 (32.90)	153 (34.23)	53 (60.92)	247 (40.83)	226 (28.75)	473 (34.00)
Lost to follow-up	0 (0.00)	0 (0.00)	0 (0.00)	0 (0.00)	0 (0.00)	5 (0.83)	2 (0.25)	7 (0.50)

*ALD 30, 31, or 32 per French Healthcare System definition.

cSCIg-1: Gammanorm^®^; cSCIg-2: Hizentra^®^; fSCIg: HyQvia^®^.

ALD, long-term disease; ATC, Anatomical Therapeutic Chemical; CMU-C, complementary universal health coverage; PID, primary immunodeficiency; SID, secondary immunodeficiency; SD, standard deviation.

#### SID population

Mean age of fSCIg- and cSCIg-treated patients was 68.1 vs 64.9 years, respectively, and 46.0% vs 48.7% of patients were female (Table 1). The most frequent comedications were those treating digestive and metabolic disorders (58.6% vs 56.7%) and systemic antibacterials (47.1% vs 47.5%), and the most common comorbidities were cancer (83.9% vs 78.8%), respiratory disorders (43.7% vs 33.8%), and hypertension (23.0% vs 31.1%) in fSCIg- vs cSCIg-treated patients, respectively. Mean±SD follow-up duration was lower in fSCIg-treated patients (7.5±4.6 months) than in cSCIg-treated patients (8.4±6.7 months). The main reason for follow-up censoring was end of follow-up (60.9%) in fSCIg-treated and treatment discontinuation (51.2%) in cSCIg-treated patients.

### HCRU and costs associated with SCIg administration

#### PID population

**Fig 3:**
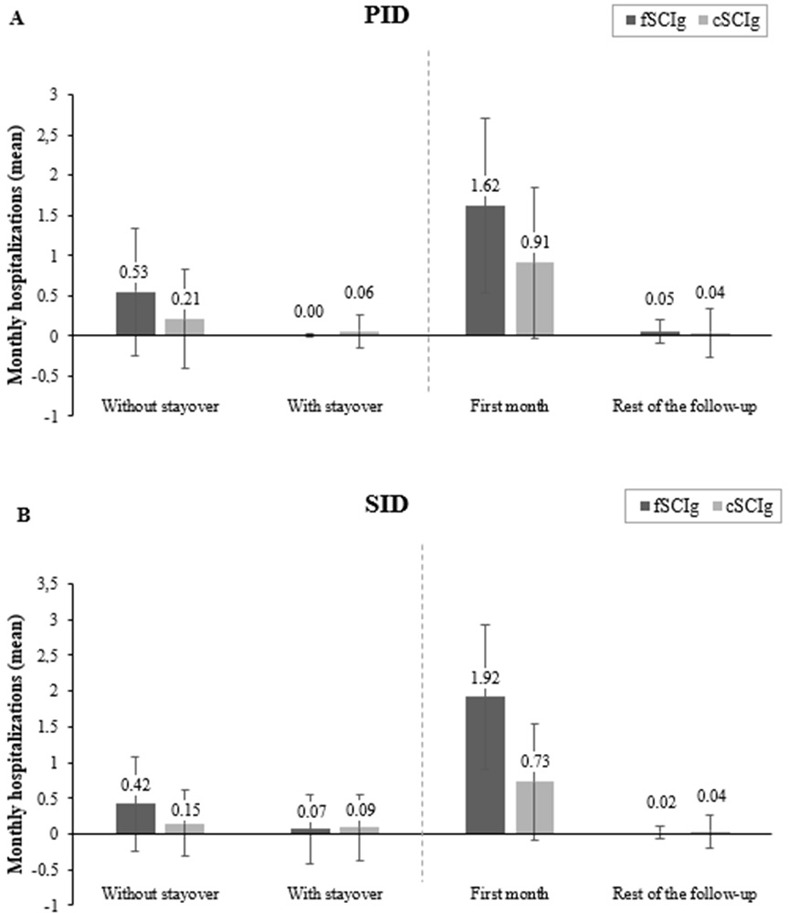
HCRU for SCIg administration-related hospitalization. (**A**) Mean monthly hospitalizations in patients with PID and (**B**) mean monthly hospitalizations in patients with SID Data are presented as mean and error bars represent SD cSCIg, conventional SCIg; fSCIg, facilitated SCIg; HCRU, healthcare resource utilization; PID, primary immunodeficiency; SCIg, subcutaneous immunoglobulin; SD, standard deviation; SID, secondary immunodeficiency.

Mean monthly number of hospitalizations (Table 2) was lower in cSCIg- vs fSCIg-treated patients (RR: 0.52; 95% CI: 0.44–0.63). Regardless of the treatment subgroup, a) the mean monthly number of hospitalizations for SCIg administration without stayover was greater than that with stayover; and b) the monthly number of hospitalizations for SCIg administration was greater within the first month of follow-up than during the rest of the follow-up period (Fig 3A). For other hospitalizations not related to SCIg administration, see (S1 Table) in the Online Repository at www.jacionline.org.

**Table 2:** HCRU and costs related to SCIg administration in patients with PID.

Monthly variables		Descriptive, nonadjusted results, mean±SD				Adjusted results, RR (95% CI)
		fSCIg	cSCIg-1	cSCIg-2	cSCIg	cSCIg vs fSCIg
		n = 87	n = 216	n = 231	n = 447	n = 534
**HCRU**	SCIg hospitalization	0.55±0.79	0.32±0.59	0.32±0.73	0.32±0.66	0.52 (0.44–0.63)
	PPV	2.60±2.58	6.47±3.39	6.40±3.35	6.44±3.37	3.06 (2.91–3.22)
	NV	1.28±1.87	3.54±4.93	3.25±4.70	3.39±4.81	2.46 (2.29–2.63)
	SCIg (≥8 g)	26.75±9.67*	21.48±11.58^†^	24.35±12.80^‡^	23.00±12.30^§^	−4.04 (−6.90, −1.17)
**Costs (€2019)**	SCIg hospitalization	243±432	662±3,823	498±1,940	577±2,999	1.63 (1.26–2.10)
	PPV	261±362	582±255	573±329	578±295	2.38 (1.87–3.02)
	NV	42±70	144±169	127±255	135±218	2.58 (2.02–3.29)
	SCIg (≥8 g)	1,245±450	1,007±537	1,135±599	1,079±573	0.71 (0.56–0.90)

*n = 81

^†^n = 157

^‡^n = 176

^§^n = 333.

cSCIg-1: Gammanorm^®^; cSCIg-2: Hizentra^®^; fSCIg: HyQvia^®^.

CI, confidence interval; HCRU, healthcare resource utilization; NV, nurse visits; PID, primary immunodeficiency; PPV, pump provider visits; RR, relative risk; SCIg, subcutaneous immunoglobulin; SD, standard deviation.

cSCIg- treated patients’ mean monthly number of PPV and NV was around 2.5 to 3 times higher compared to fSCIg-treated ones (RR: 3.06; 95% CI: 2.91–3.22 and RR: 2.46; 95% CI: 2.91–3.22, respectively). SCIg doses delivered in hospital or at home were around 4 grams lower in cSCIg- vs fSCIg-treated patients (average difference: −4.04; 95% CI: −6.90, −1.17)

In cSCIg- vs fSCIg-treated patients, respectively, mean monthly costs (Table 2) were around 1.5 higher for hospital SCIg administration (€577 vs €243; RR: 1.63; 95% CI: 1.26–2.10), 2.5 higher for PPV (€578 vs €261; RR: 2.38; 95% CI: 1.87–3.02), and for NV (€135 vs €42; RR: 2.58; 95% CI: 2.02–3.29). Mean monthly cost of SCIg doses (≥8 g/month) was 30% lower in cSCIg- vs fSCIg-treated patients (€1,079 vs €1,245, respectively; RR: 0.71; 95% CI: 0.56–0.90).

#### SID population

Mean monthly number of hospitalizations (Table 3) was two times lower in cSCIg- vs fSCIg-treated patients (RR: 0.43; 95% CI: 0.36–0.50). Regardless of the treatment subgroup, a) the mean monthly number of hospitalizations for SCIg administration without stayover was greater than that with stayover and was greater within the first month of follow-up than during the rest of the follow-up period and b) the monthly number of hospitalizations for SCIg administration was greater within the first month of follow-up than during the rest of the follow-up period (Fig 3B). For other hospitalizations not related to SCIg administration, see (S1 Table) in the Online Repository at www.jacionline.org.

**Table 3:** HCRU and costs related to an SCIg administration in patients with SID.

Monthly variables		Descriptive, nonadjusted results, mean±SD				Adjusted results, RR (95% CI)
		fSCIg	cSCIg-1	cSCIg-2	cSCIg	cSCIg vs fSCIg
		n = 87	n = 605	n = 786	n = 1,391	n = 1,478
**HCRU**	SCIg hospitalization	0.53±0.79	0.30±0.66	0.31±0.66	0.30±0.66	0.43 (0.36–0.50)
	PPV	2.09±1.74	5.39±3.25	5.90±4.66	5.61±3.94	3.09 (2.92–3.28)
	NV	3.06±5.07	4.24±5.24	4.62±5.58	4.41±5.39	1.56 (1.49–1.64)
	SCIg (≥8 g)	23.44±8.42*	23.25±15.94^†^	22.74±16.29^‡^	23.05±16.07^§^	−0.71 (−4.38, 2.97)
**Costs (€2019)**	SCIg hospitalization	475±1,662	2,079±22,877	1,296±6,012	1,739±17,647	1.76 (1.40–2.21)
	PPV	226±264	488±278	548±438	514±358	2.23 (1.79–2.76)
	NV	60±65	149±152	169±174	158±162	2.61 (2.09–3.25)
	SCIg (≥8 g)	1,090±392	1,079±744	1,064±773	1,073±755	0.82 (0.66–1.02)

*n = 75

^†^n = 597

^‡^n = 382

^§^n = 979.

cSCIg-1: Gammanorm^®^; cSCIg-2: Hizentra^®^; fSCIg: HyQvia^®^.

CI, confidence interval; HCRU, healthcare resource utilization; NV, nurse visits; PPV, pump provider visits; RR, relative risk; SCIg, subcutaneous immunoglobulin; SD, standard deviation; SID, secondary immunodeficiency.

cSCIg- treated patients’s mean monthly number of PPV and NV (Table 3) was around 1.5 to 3 times higher compared to fSCIg-treated (RR: 3.09; 95% CI: 2.92–3.28 and RR: 1.56; 95% CI: 1.49–1.64, respectively). SCIg doses delivered in hospital or at home were not different in cSCIg- vs fSCIg-treated patients (average difference: −0.71; 95% CI: −4.38, 2.97).

In cSCIg- vs fSCIg-treated patients, respectively, mean monthly costs (Table 3) were 1.7 higher for hospital SCIg administration (€1,739 vs €475; RR: 1.76; 95% CI: 1.40–2.21), 2.2 times higher for PPV (€514 vs €226; RR: 2.23; 95% CI: 1.79–2.76), and 2.6 time higher for NV (€158 vs €60; RR: 2.61; 95% CI: 2.09–3.25). Mean monthly cost of SCIg doses was not different in cSCIg- vs fSCIg-treated patients (€1,073 vs €1,090; RR: 0.82; 95% CI: 0.66-1.02).

## Discussion

This real-world study compared the HCRU and associated costs for fSCIg with those for cSCIg for the treatment of PID and SID in France and suggests that patients treated with fSCIg had fewer at-home HCRU and lower overall costs for in-hospital or at-home SCIg administration.

Previous studies have shown the benefits of using fSCIg [20,42,43], but real-world data on SCIg-related HCRU are limited in France. When switching from IVIg or cSCIg therapy to fSCIg therapy in patients with PID, fSCIg was shown to be clinically effective [22] and preferred to IVIg or cSCIg in Polish and American real-world studies [22,23]. Real-world experience with fSCIg in patients with PID showed that fSCIg training ramp-up time and administration specifics can be tailored to individual patient needs and preferences [44]. In a European real-word study comparing home-based, self-administered fSCIg therapy with cSCIg therapy in patients with PID and SID, a longer training period was required for fSCIg than for cSCIg; but IgG levels were stable for fSCIg training [23]. Data from other real-life studies conducted in other countries showed the effectiveness, safety, and tolerability of fSCIg in patients with PID and SID, but did not compare HCRU and costs with that in patients receiving cSCIg [26,45,46].

In the current study, the PID and SID cohorts had comparable baseline characteristics and expected clinical profiles. fSCIg-treated patients had slightly fewer comorbidities at treatment initiation than cSCIg-treated patients, indicating that they may have a more favorable clinical profile compared with cSCIg-treated patients.

Studies have reported that HCRU and costs are markedly lower for SCIg vs IVIg administration [43,47], and hospitalizations due to infections are low in patients receiving fSCIg treatment [22,42]. In this study, cSCIg-treated patients had lower hospitalization rates related to Ig administration but higher at-home HCRU than fSCIg-treated patients in the PID and SID cohorts. Notably, most administration-related hospitalizations were without stayover and occurred during the first month of follow-up. Patients receiving fSCIg likely visit the hospital once weekly during the first two infusions for ramp-up and dose adjustments, followed by receiving at-home infusions once a month. This could, to a large extent, explain the high hospitalization rates without stayover occurring in the first month of follow-up in fSCIg-treated patients reported in this study. Our findings correspond to the clinical practice requiring a typical ramp-up period after fSCIg initiation for dose adjustment [23,44].

In this study, mean monthly utilization of at-home PPV and NV was 1.5–3 times higher with cSCIg than with fSCIg treatment in both the PID and SID cohorts. The high number of NV across treatment cohorts was most likely related to the lack of specificity of the codes used to identify NV and exclusion of NV unrelated to SCIg administration. Thus, the rate of self-administration of SCIg was difficult to assess. Regardless of in-hospital or at-home administration, mean monthly SCIg doses were lower with cSCIg vs fSCIg treatment.

To date, no studies have been published on the costs of cSCIg and fSCIg. Although several studies suggest that SCIg may be less costly than IVIg and could potentially improve QUALYs, the evidence remains limited and context-dependent [48–50]. For example, a recent Canadian study found that the average per patient-year cost was 5,386 $CDN (3625 €2019) lower for self-administered SCIg versus clinic-administered IVIg [51]. Similarly, an Australian study reported an average annual cost difference of 5,583 $AUD (3,467 €2019) per patient, with an approximate annual cost of SCIg of 25,171$AUD (15,631 €2019) per patient [48]. However, it should be noted that the latter conclusions were based on a Markov model with a small cohort of 14 PID patients, limiting the generalizability of the findings In Spain, a 2022 study focusing on PID patients found a difference of 4,266€ between IVIg and SCIg, with an annual costs of 14,466 € for SCIg treatment [52]. These findings are consistent with our results, where the mean monthly cost of SCIg doses ranged between €1,079 for cSCIg and €1,245 for fSCIg (annual average of 12,948€ and 14,940€, respectively). However, while these data suggest a cost advantage for SCIg in specific contexts, more comprehensive, real-world studies are needed to confirm these trends.

The SNDS database covers all reimbursed healthcare consumption in France. As such, all HCRU-related costs assessed in this study were reimbursable healthcare consumptions, which are important in the management of immunodeficient patients in France. Mean monthly costs of in-hospital and at-home (PPV and NV) SCIg administration were on average 1.6–1.8 and 2.2–2.6 times higher, respectively, with cSCIg vs fSCIg treatment in the PID and SID cohorts. The costlier results observed for at-home PPV (with or without NV) within patients receiving once weekly cSCIg than for those receiving once monthly fSCIg, can be explained by the frequency of administration. However, mean monthly cost of SCIg doses was slightly lower (0.7–0.8 times) with cSCIg vs fSCIg treatment. The overall results indicate a lower cost burden in the management of patients with PID or SID receiving fSCIg vs that of patients receiving cSCIg, irrespective of in-hospital or at-home administration. Cost differences between patients receiving fSCIg and those receiving cSCIg were prominent for infusion pumps within the total cost, indicating that infusion pumps were one of the major cost drivers. Irrespectively of tariff rates, given the frequency of administration, the costs of pump service providers were higher in cSCIg- vs fSCIg-treated patients (see (S1 Table) in Online Repository at www.jacionline.org).

The main strength of this study was that the patient data were collected from the SNDS, a national representative administrative data source. To our knowledge, no real-life study assessing SCIg-replacement therapy in patients with PID or SID has been conducted to date using data from this comprehensive database. In general, PIDs are underdiagnosed in most countries, making it difficult to collect sufficient data on the morbidity and mortality of PID [6]. SNDS, on the other hand, covers around 99% of the French population (over 66 million individuals), with routine and updated information on prescriptions, patients, practitioners, and pharmacies [32,33]; thus, the data collected represented an almost complete population of patients with diagnosed PID or SID.

Our study had some limitations. The SNDS database does not contain medical results, including body weight, laboratory tests or imaging results; therefore, data for the dose in gram per kilogram of bodyweight could not be described. Besides, the current ICD-10 codes do not accurately identify patients with all SID conditions, due to which they were identified by exclusion of patients with PID leading to probable misclassification of some patients with PID. PID population selection may also have been biased by the inclusion of a few possible patients with SID, as a higher percentage than expected was observed for patients with cancer (in regard to a low rate of antineoplastic agents’ administration), which may be due to some inaccurate coding of chronic diseases in the SNDS database. Additionally, inclusion criteria of patients not having received Ig in the prior 6 months might have led to misclassification of newly treated patients. Although fSCIg and cSCIg cohorts were similar in their baseline characteristics, there was no comparison of possible clinical differences. Differences in hospitalizations between fSCIg- and cSCIg-treated patients could not be fully explained due to the lack of detailed clinical information or miscoding of the diagnosis as some patients with PID also had SID-related codes. Models took into account the statistical significant covariates to adjust on patients characteristics, however external factors not reachable in the database could influence the results. Moreover, data were not confounded by the type of indication to compare the characteristics of patients with PID with those of patients with SID. Although overall treatment discontinuation rates were high, a lower percentage of fSCIg-treated patients discontinued treatment compared with cSCIg-treated patients in both the PID and SID cohorts. The high rate of discontinuations could be due to the algorithm used to identify discontinuation in the SNDS database, or due to potential seasonal use of Ig (while this is not a recommended practice) and long holiday periods between subsequent treatments (while alternative route should be considered with the practitioner), or finally, because of poor tolerance of the SC route. However, the algorithm did not identify any switch to another route in these patients.

In conclusion, this study provides real-world evidence on characteristics and HCRU in patients with PID and SID in France, confirming the known clinical profile of these patients including comorbidities. fSCIg- vs cSCIg-treated patients had higher hospitalization rates for Ig adminstrations but lower at-home HCRU in the PID and SID cohorts. The higher hospitalizations rates without stayover in the fSCIg subgroup can be explained by the dose adjustments necessary during treatment initiation. Mean monthly utilization of PPV and NV was higher in patients receiving weekly cSCIg vs monthly fSCIg, while the SCIg doses utilized were slightly higher in patients receiving fSCIg vs cSCIg. Regardless of the indication, fSCIg-treated patients had lower HCRU-related costs for SCIg-administration vs cSCIg-treated patients. Overall, fSCIg presents a suitable treatment option with reduced HCRU and healthcare-related costs.

## Supporting information

S1 Table Other HCRU during the follow-up period in patients with PID. *Include medical care, care involving dialysis, need for other prophylactic measures, transplanted organ and tissue status, and follow-up examination after treatment for conditions other than malignant neoplasms; ^†^Mainly include nurses, physiotherapists, and general practitioners cSCIg-1: Gammanorm^®^; cSCIg-2: Hizentra^®^; fSCIg: HyQvia^®^ HCRU, healthcare resource utilization; fSCIg, facilitated immunoglobulin, immunoglobulin; PID, primary immunodeficiency; SD, standard deviation.
(DOCX)

S2 Table Other HCRU during the follow-up period in patients with SID. *Include medical care, care involving dialysis, need for other prophylactic measures, transplanted organ and tissue status, and follow-up examination after treatment for conditions other than malignant neoplasms; ^†^Mainly include nurses, physiotherapists, and general practitioners cSCIg-1: Gammanorm^®^; cSCIg-2: Hizentra^®^; fSCIg: HyQvia^®^ HCRU, healthcare resource utilization; Ig, immunoglobulin; SD, standard deviation; SID, secondary immunodeficiency.
(DOCX)

S3 Table PERFADOM costs recalculated with the most recent tariff. The price application start date was June 27, 2019, according to the reference price as: PERFADOM 1, €357.20; PERFADOM 7, €100.75; PERFADOM 10, €35.72; PERFADOM 25, €49.28 PID, primary immunodeficiency; SD, standard deviation; SID, secondary immunodeficiency.
(DOCX)

S1 Fig Definition of follow-up based on treatment exposure time. FU, follow-up; IVIg, intravenous immunoglobulin; PID, primary immunodeficiency; SCIg, subcutaneous immunoglobulin; SID, secondary immunodeficiency.
(DOCX)

## Abbreviations

ALD: Affections de longue durée
ATC: Anatomical Therapeutic Chemical
CEREDIH: Centre de Référence Déficits Immunitaires Héréditaires
CESREES: Comité éthique et scientifique pour les recherches, les études et les évaluations dans le domaine de la santé
CI: Confidence interval
CMU-C: Couverture Maladie Universelle Complémentaire
CNIL: Commission nationale de l’informatique et des libertés
cSCIg: Conventional subcutaneous immunoglobulin
DCIR: Données de consommation inter-régimes
ESID: European Society for Immunodeficiencies
fSCIg: Facilitated subcutaneous immunoglobulin
HCRU: Healthcare resource utilization
ICD-10: International Classification of Disease, Tenth Revision
Ig: Immunoglobulin
IVIg: Intravenous immunoglobulin
MA: Marketing authorization
PID: Primary immunodeficiency
PMSI: Programme de Médicalisation des Systèmes d’Information
RR: Relative risk
SCIg: Subcutaneous immunoglobulin
SD: Standard deviation
SID: Secondary immunodeficiency
SNDS: Système national des données de santé
SNIIRAM: Système national d’information interrégimes de l’Assurance Maladie
