# Supplementary material for: Healthcare resource utilization and costs in immunodeficient patients receiving subcutaneous Ig: Real-world evidence from France
Source: PLoS One. 2025 Jan 24;20(1):e0313694. doi: 10.1371/journal.pone.0313694 (PMC11759344; doi:10.1371/journal.pone.0313694)
Supplement: S1 Table — *Include medical care, care involving dialysis, need for other prophylactic measures, transplanted organ and tissue status, and follow-up examination after treatment for conditions other than malignant neoplasms; †Mainly include nurses, physiotherapists, and general practitioners cSCIg-1: Gammanorm®; cSCIg-2: Hizentra®; fSCIg: HyQvia® HCRU, healthcare resource utilization; fSCIg, facilitated immunoglobulin, immunoglobulin; PID, primary immunodeficiency; SD, standard deviation. (DOCX) [file pone.0313694.s001.docx]

**S1 - Supplemental material****, Online repository**

**S1 Table E1.** Other HCRU during the follow-up period in patients with PID

| **HCRU (monthly number), mean±SD** | fSCIg | cSCIg-1 | cSCIg-2 | cSCIg |
| --- | --- | --- | --- | --- |
|  | **n=87** | **n=216** | **n=231** | **n=447** |
| Hospitalizations without Ig administration* | 3.59±9.65 | 3.60±7.16 | 4.98±11.36 | 4.31±9.58 |
| Deliveries for treatment | 0.95±0.86 | 1.14±0.98 | 1.13±0.96 | 1.13±0.97 |
| Laboratory tests | 18.03±40.45 | 18.68±30.95 | 25.67±50.94 | 22.29±42.57 |
| Imaging procedures | 1.10±2.74 | 1.21±2.39 | 1.44±3.89 | 1.73±3.87 |
| Central venous catheters | 0.05±0.22 | 0.10±0.33 | 0.17±0.73 | 0.16±0.63 |
| Sick leaves | 0.03±0.14 | 0.02±0.09 | 0.01±0.04 | 0.01±0.07 |
| Professional visits^†^ | 48.52±151.33 | 96.08±394.18 | 109.68±331.79 | 103.11±362.93 |

*Include medical care, care involving dialysis, need for other prophylactic measures, transplanted organ and tissue status, and follow-up examination after treatment for conditions other than malignant neoplasms; ^†^Mainly include nurses, physiotherapists, and general practitioners

cSCIg-1: Gammanorm^®^; cSCIg-2: Hizentra^®^; fSCIg: HyQvia^®^

HCRU, healthcare resource utilization; fSCIg, facilitated immunoglobulin, immunoglobulin; PID, primary immunodeficiency; SD, standard deviation
