# Supplementary material for: Healthcare resource utilization and costs in immunodeficient patients receiving subcutaneous Ig: Real-world evidence from France
Source: PLoS One. 2025 Jan 24;20(1):e0313694. doi: 10.1371/journal.pone.0313694 (PMC11759344; doi:10.1371/journal.pone.0313694)
Supplement: S2 Table — *Include medical care, care involving dialysis, need for other prophylactic measures, transplanted organ and tissue status, and follow-up examination after treatment for conditions other than malignant neoplasms; †Mainly include nurses, physiotherapists, and general practitioners cSCIg-1: Gammanorm®; cSCIg-2: Hizentra®; fSCIg: HyQvia® HCRU, healthcare resource utilization; Ig, immunoglobulin; SD, standard deviation; SID, secondary immunodeficiency. (DOCX) [file pone.0313694.s002.docx]

**S1 - Supplemental material, Online repository**

**S1 Table E2.** Other HCRU during the follow-up period in patients with SID

| **HCRU (monthly number), mean±SD** | fSCIg | cSCIg-1 | cSCIg-2 | cSCIg |
| --- | --- | --- | --- | --- |
|  | **n=87** | **n=605** | **n=786** | **n=1,391** |
| Hospitalizations without Ig administration* | 6.56±23.51 | 5.68±19.31 | 6.55±20.47 | 6.17±19.97 |
| Deliveries for treatment | 1.38±1.10 | 1.43±1.28 | 1.29±1.12 | 1.35±1.19 |
| Laboratory tests | 39.00±110.15 | 31.20±72.33 | 35.48±95.00 | 33.62±85.88 |
| Imaging procedures | 2.18±10.66 | 2.36±8.95 | 1.77±5.31 | 2.03±7.13 |
| Central venous catheters | 0.17±1.09 | 0.20±0.88 | 0.29±1.87 | 0.25±1.52 |
| Sick leaves | 0.01±0.04 | 0.02±0.12 | 0.01±0.03 | 0.01±0.08 |
| Professional visits^†^ | 88.61±227.56 | 78.76±278.26 | 100.80±267.85 | 91.22±272.55 |

*Include medical care, care involving dialysis, need for other prophylactic measures, transplanted organ and tissue status, and follow-up examination after treatment for conditions other than malignant neoplasms; ^†^Mainly include nurses, physiotherapists, and general practitioners

cSCIg-1: Gammanorm^®^; cSCIg-2: Hizentra^®^; fSCIg: HyQvia^®^

HCRU, healthcare resource utilization; Ig, immunoglobulin; SD, standard deviation; SID, secondary immunodeficiency
