# Supplementary material for: Healthcare resource utilization and costs in immunodeficient patients receiving subcutaneous Ig: Real-world evidence from France
Source: PLoS One. 2025 Jan 24;20(1):e0313694. doi: 10.1371/journal.pone.0313694 (PMC11759344; doi:10.1371/journal.pone.0313694)
Supplement: S3 Table — The price application start date was June 27, 2019, according to the reference price as: PERFADOM 1, €357.20; PERFADOM 7, €100.75; PERFADOM 10, €35.72; PERFADOM 25, €49.28 PID, primary immunodeficiency; SD, standard deviation; SID, secondary immunodeficiency. (DOCX) [file pone.0313694.s003.docx]

**S1 - Supplemental material, Online repository**

**S1 Table E3**. PERFADOM costs recalculated with the most recent tariff

| **PERFADOM monthly cost (**€**)** | | | **Descriptive, nonadjusted results, mean±SD** | | |
| --- | --- | --- | --- | --- | --- |
|  | HyQvia | Gammanorm | | Hizentra | Gammanorm or Hizentra |
| PID | 254.91±360.87 | 535.76±251.84 | | 523.53±292.36 | 529.44±273.29 |
| SID | 210.19±252.61 | 484.07±291.29 | | 452.17±277.85 | 466.05±284.11 |

The price application start date was June 27, 2019, according to the reference price as: PERFADOM 1, €357.20; PERFADOM 7, €100.75; PERFADOM 10, €35.72; PERFADOM 25, €49.28

PID, primary immunodeficiency; SD, standard deviation; SID, secondary immunodeficiency
