# Supplementary material for: Healthcare resource utilization and costs in immunodeficient patients receiving subcutaneous Ig: Real-world evidence from France
Source: PLoS One. 2025 Jan 24;20(1):e0313694. doi: 10.1371/journal.pone.0313694 (PMC11759344; doi:10.1371/journal.pone.0313694)
Supplement: S1 Fig — FU, follow-up; IVIg, intravenous immunoglobulin; PID, primary immunodeficiency; SCIg, subcutaneous immunoglobulin; SID, secondary immunodeficiency. (DOCX) [file pone.0313694.s004.docx]

**S1 - Supplemental material, Online repository**

**S1 FIG E1.** Definition of follow-up based on treatment exposure time


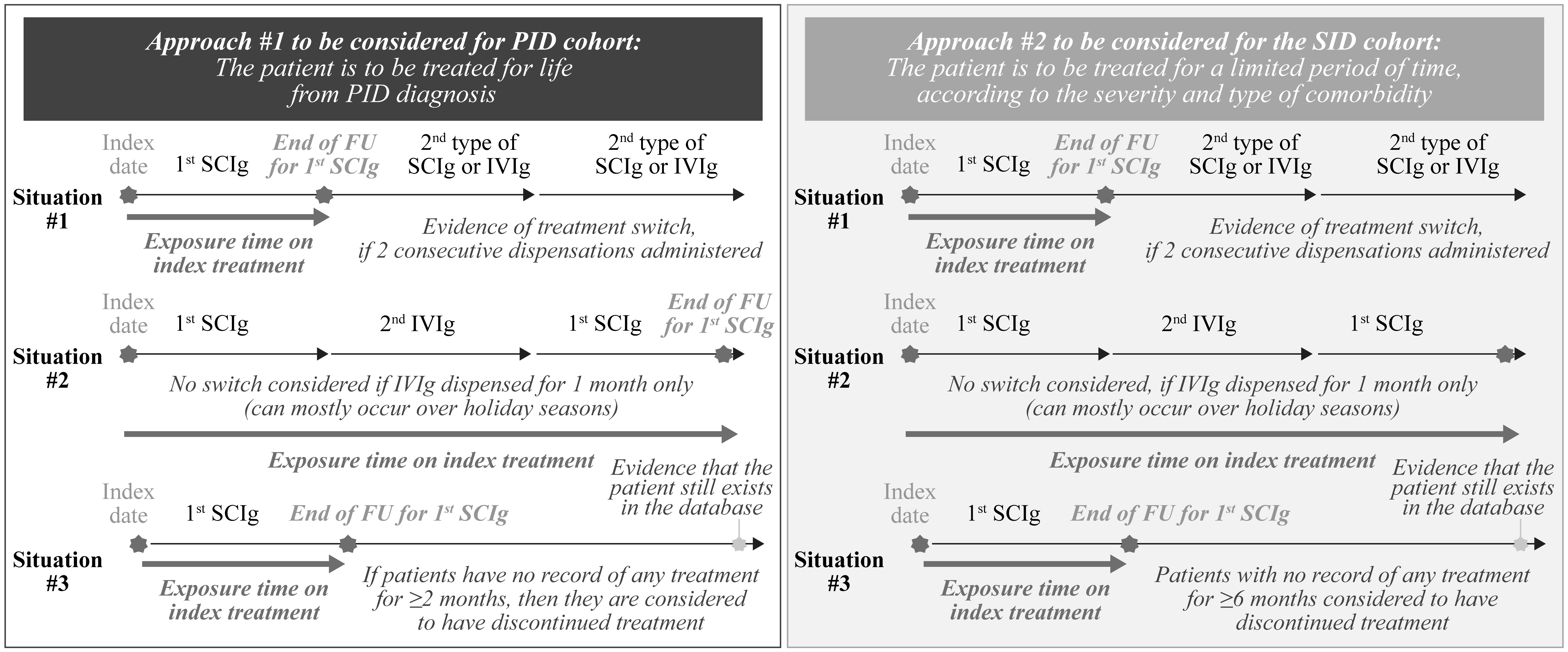


FU, follow-up; IVIg, intravenous immunoglobulin; PID, primary immunodeficiency; SCIg, subcutaneous immunoglobulin; SID, secondary immunodeficiency
